# Thermal runaway of Lithium-ion batteries employing LiN(SO_2_F)_2_-based concentrated electrolytes

**DOI:** 10.1038/s41467-020-18868-w

**Published:** 2020-10-09

**Authors:** Junxian Hou, Languang Lu, Li Wang, Atsushi Ohma, Dongsheng Ren, Xuning Feng, Yan Li, Yalun Li, Issei Ootani, Xuebing Han, Weining Ren, Xiangming He, Yoshiaki Nitta, Minggao Ouyang

**Affiliations:** 1 State Key Laboratory of Automotive Safety and Energy, Tsinghua University, 100084 Beijing, China; 2 Institute of Nuclear and New Energy Technology, Tsinghua University, 100084 Beijing, China; 3 Advanced Materials and Processing Laboratory, Nissan Motor Co., Ltd., 1, Natsushima-cho, Yokosuka, 237-8523 Japan

**Keywords:** Chemical safety, Batteries, Batteries

## Abstract

Concentrated electrolytes usually demonstrate good electrochemical performance and thermal stability, and are also supposed to be promising when it comes to improving the safety of lithium-ion batteries due to their low flammability. Here, we show that LiN(SO_2_F)_2_-based concentrated electrolytes are incapable of solving the safety issues of lithium-ion batteries. To illustrate, a mechanism based on battery material and characterizations reveals that the tremendous heat in lithium-ion batteries is released due to the reaction between the lithiated graphite and LiN(SO_2_F)_2_ triggered thermal runaway of batteries, even if the concentrated electrolyte is non-flammable or low-flammable. Generally, the flammability of an electrolyte represents its behaviors when oxidized by oxygen, while it is the electrolyte reduction that triggers the chain of exothermic reactions in a battery. Thus, this study lights the way to a deeper understanding of the thermal runaway mechanism in batteries as well as the design philosophy of electrolytes for safer lithium-ion batteries.

## Abstract (web summary)

Concentrated electrolytes display superior thermal stability due to their non-flammability nature. Here, the authors show that LiN(SO_2_F)_2_-based concentrated electrolytes are incapable of solving the safety issues due to heat release during reaction between the lithiated graphite and electrolyte.

## Introduction

The application of large-format lithium-ion batteries (LIBs) with high energy density in electric vehicles requires a high level of battery safety^1–9^, as the fires and explosions of batteries that take place during thermal runaway pose serious threats to the lives of passengers and property^1,10–12^. Thermal runaway is driven by a chain of exothermic reactions that spontaneously increase the temperature of lithium-ion batteries. As a result, severe redox exothermic reactions are likely triggered at relatively high temperatures, generating a tremendous amount of heat and leading to uncontrollable rise in temperature^11–19^. Thus, the removal or reduction of the main exothermic reactions during the evolution of thermal runaway is essential to guarantee the safety of lithium-ion batteries.

Flammable carbonate-based electrolytes have been widely used in commercial LIBs, and they are considered to be responsible for thermal runaway^2,3,20^, which is reasonable, as they are among the main fuels that increase vigorous combustion. Thus, non-flammable electrolytes are considered and thought to improve battery safety. Concentrated electrolytes and localized or aqueous concentrated electrolytes have been receiving considerable attention in recent years, due to their high electrochemical performance, low volatility, and low flammability^20–27^. Many fundamental studies revealed that the unique solution structure, where all the solvent molecules and even anions are involved in the solvation sheath, endow concentrated electrolytes with different properties in comparison with the same compositional but traditional 1 M electrolytes. For example, in a battery, when the molar ratio of lithium bisimide (fluorosulfonyl) (LiN(SO_2_F)_2_, LiFSI) to the solvent is 1:1.9, the predominant contact ion pairs (CIPs, an anion coordinating to one Li^+^) and aggregates (AGGs, an anion coordinating to two or more Li^+^) in the solvated structures guarantee the stable cycling and improve thermal properties of LIBs^22,28,29^. Meanwhile, fire retardant solvents, such as trimethyl phosphate (TMP), triethyl phosphate (TEP), and trisphosphate (trifluoroethyl) (TFEP), can be employed to develop non-flammable electrolytes due to the changes in the interface reactions that are led by the unique solvation structure of concentrated electrolytes^20,24,30^. However, although the high thermal stability of concentrated electrolytes, which enhances battery safety as implied by the researchers, has been widely proven by ignition tests and thermogravimetric analyses (TGA)^20,24,29^. No measurements using a practical battery have been reported to directly verify the improved safety of LIBs with advanced concentrated electrolytes. It has been reported that the highly energetic thermal runaway may release only a modest amount of heat in flaming combustion and vice versa^31,32^.

Thus, a direct measurement of the safety of batteries with concentrated electrolytes is necessary to clarify the truth. In addition, the proposed mechanism will deepen our understanding of thermal runaway. Generally, it is understandable that stopping the triggered redox exothermic reactions can help reduce the destructiveness of battery thermal runaway, which can be achieved by, for example, replacing the layered cathode material with LiFePO_4_. Moreover, avoiding the initial triggers or shutting down the reaction chain is very meaningful for safety control. For most of the battery chemistries with layered oxide cathodes and graphite anodes, such as graphite|LiNi_0.3_Co_0.3_Mn_0.3_O_2_ (Gr|NMC333) batteries, it was confirmed that the heat generated before 250 °C is dominated by the reactions between the anode and the electrolyte, which is believed to extremely increase the battery’s temperature to a level that initiates the final thermal runaway reactions^7,33,34^. Besides, the chemical crosstalk in graphite|LiNi_0.5_Mn_0.3_ Co_0.2_O_2_ (Gr|NMC532) batteries was proven to trigger thermal runaway without internal short circuits (ISC)^12^. In addition, the lithiated anode consumes the highly oxidative gases released during the cathode phase transition, thus producing tremendous heat that brings the battery to the thermal runaway point^12^. In this case, the electrolyte is a dispensable ingredient for thermal runaway. Roughly, the exothermic reactions inside the battery can be divided into three groups: the reactions between the anode-electrolyte (AnEly), the reactions between the cathode-electrolyte (CaEly), and the reactions between the cathode-anode (CaAn)^11^. Among these reactions, the AnEly reactions contribute to the initial heat accumulation, and the CaEly and CaAn reactions both result in drastic combustion, but they need a considerable high temperature to start^11,12,33^. Considering that non-flammability is an essential result of the CaEly reactions, predicting the safety of a charged battery is insufficient.

In this work, we employ accelerated rate calorimetry (ARC), differential scanning calorimeter (DSC) coupled with thermal gravimetric analysis (TGA), and mass spectrometry (DSC-TG-MS) experiments to evaluate the safety performance of graphite|LiNi_0.8_Co_0.1_Mn_0.1_O_2_ (Gr|NMC811) and Gr|NMC532 batteries with concentrated electrolytes. The thermal runaway mechanism at both the cell and material level is systematically investigated. The most promising concentrated electrolytes, LiFSI/DMC (1:1.9 by molar) and LiFSI/TMP (1:1.9 by molar), are selected in this study. The results show that the non-flammable concentrated electrolytes could not prevent LIBs from thermal runaway. Specifically, the charged anode reacts with LiFSI and releases a considerable amount of heat that triggered thermal runaway.

## Results

### Cycling and thermal properties of the electrolytes

**Fig. 1:**
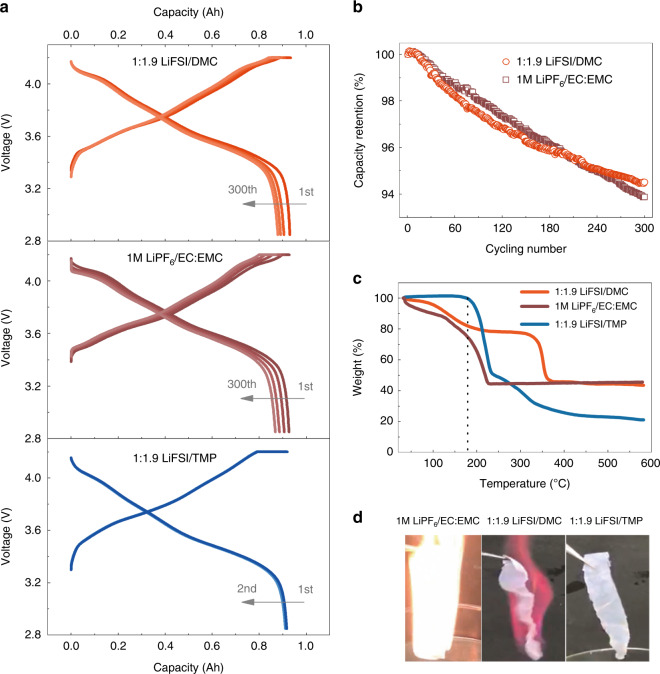
Electrochemical performance and physical properties of concentrated electrolytes. **a** Charge and discharge plots of the Gr|NMC811 battery with LiFSI/DMC (1:1.9 by molar), LiFSI/TMP (1:1.9 by molar), and conventional 1 M LiPF_6_/EC:EMC (3:7 by volume). All batteries delivered a reversible capacity of 0.93 Ah, which was approximated to the design capacity of 0.95 Ah. **b** Cycling performance of Gr|NMC811 battery with LiFSI/DMC concentrated electrolyte and 1 M LiPF_6_/EC:EMC. **c** TGA curves showed the weight loss of the LiFSI/DMC and LiFSI/TMP concentrated electrolytes and the 1 M LiPF_6_/EC:EMC electrolyte. **d** Flammability of the LiFSI/DMC and LiFSI/TMP concentrated electrolytes and the 1 M LiPF_6_/EC:EMC electrolyte. Ignition tests were performed using polyethylene separators, which were saturated with the electrolytes. A flame igniter produced a flame with a temperature of above 1400 °C. The photos displayed the moment when the electrolytes were burning with the most vigorous flame.

Figure 1a shows that the 0.93Ah Gr|NMC811 pouch cell with the concentrated LiFSI/DMC electrolyte delivered stable charge-discharge capacities for over 300 cycles at C/3. The average coulombic efficiency was 96.6% and capacity retention was 94.5% (Fig. 1b), indicated the suppressed Al dissolution and stable SEI on the graphite during cycling^20,24,29^. The cell with conventional 1 M LiPF_6_/EC:EMC (3:7 by volume) electrolyte shows comparable electrochemical performances, the capacity retention after 300 cycles was 93.9%. The LiFSI/TMP concentrated electrolyte in the pouch cell was also investigated for two cycles before safety evaluation, and it delivered a coulombic efficiency of 99.5% (Fig. 1a). For the Gr|NMC532 pouch cells, the charge and discharge curves also demonstrated stable electrochemical performance with the concentrated electrolytes (Supplementary Fig. 1 and Supplementary Note 1).

The TGA curves (see Fig. 1c) show that the weight loss of the LiFSI/TMP concentrated electrolyte was only 0.7 wt% below 180 °C, which is considerably lower than that of the LiFSI/DMC electrolyte (18.2 wt%) and the diluted carbonate electrolyte (26.5 wt%). These results also indicate that the flammability of the LiFSI/DMC concentrated electrolyte is lower than that of the dilute conventional electrolyte, as less solvent was used and the vitality of the DMC was significantly changed by the solvation structure. Then, Fig. 1d shows the photographs of the separators saturated with the electrolytes during the ignition test. In comparison with the conventional electrolyte, the concentrated electrolyte with the DMC was still flammable but with a mild flame, whereas the concentrated electrolyte with the self-extinguishing solvent of the TMP did not burn completely, which proves that the concentrated LiFSI/TMP is non-flammable (see details in Supplementary Table 1 and Supplementary Note 2). According to the above thermal evaluation, concentrated electrolytes display better thermal stability and possibly lower flammability than dilute electrolytes, which is in agreement with the previous reports^24,29,30^.

However, it was reported that the direct redox reactions between the charged cathode and anode are severe for battery chemistries of high energy density, which can cause thermal runaway even without electrolytes or ISC^12,13^. Thus, the evaluation of battery safety based on the used electrolytes only is not sufficient, and the interaction between the electrolytes and the charged electrodes should be systematically taken into consideration.

### Safety characterization of LiFSI/DMC in Gr|NMC batteries

**Fig. 2:**
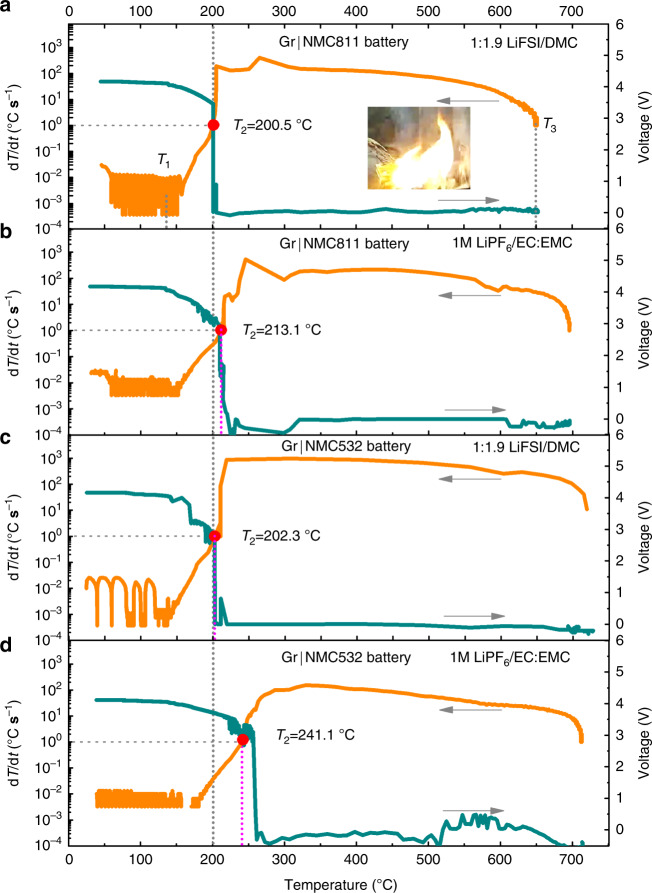
Comparison of thermal runaway features of Gr|NMC batteries with concentrated LiFSI/DMC and conventional 1 M LiPF_6_/EC:EMC electrolytes. **a** Gr|NMC811 battery with the concentrated LiFSI/DMC electrolyte. The inset shows the combustibility of battery in the lateral heating test. **b** Gr|NMC811 battery with the conventional 1 M electrolyte. **c** Gr|NMC532 battery with the concentrated LiFSI/DMC electrolyte. **d** Gr|NMC532 battery with the conventional 1 M electrolyte. d*T*/d*t*-*T* curves of Gr|NMC811 and Gr|NMC532 batteries based on the ARC test, were plotted in logarithmic coordinates. *T*_1_ was defined as the onset temperature of self-heating, which results from the onset of the chain reactions inside the battery, leads to a spontaneous and continuous rise in temperature if the battery is kept under a poor heat dissipation condition or an almost adiabatic condition. *T*_2_ was defined as the trigger temperature of thermal runaway preset at the d*T*/d*t* of 1 °C s^−1^. *T*_3_ was defined as the maximum temperature during the thermal runaway, which is a key parameter in the evaluation of the destructiveness of thermal runaway.

The thermal runaway features of Gr|NMC batteries with concentrated LiFSI/DMC and conventional 1 M LiPF_6_/EC:EMC electrolytes are compared in Fig. 2. It is observed that all the batteries were brought to the point of thermal runaway although the thermal stability of the concentrated electrolyte was obviously higher. The three characteristic temperatures of {*T*_1_, *T*_2_, *T*_3_} were defined to describe the thermal behavior of the batteries with different electrolytes^7,11,13^. In this study, in case of the concentrated and conventional electrolytes, *T*_1_ was located at ~130 °C, and *T*_3_ was between 650 °C and 730 °C, whereas *T*_2_ exhibited a totally different value (Fig. 2). *T*_2_ was defined as the trigger temperature of the thermal runaway. At and after this critical point, the battery temperature exponentially increased and could not be shut down by any heat dissipation measures. Both the severe exothermic reactions and the ISC might be the underlying reasons. If *T*_2_ is caused by a single chemical reaction or a group of chemical reactions, this or these reactions can be defined as the trigger reaction of thermal runaway. The tremendous heat generated at *T*_2_ by the trigger reaction would immediately trigger a single exothermic reaction or a group of new exothermic reactions between the battery components, causing a dramatic rise (hundreds of degrees per second) of battery temperature. Understanding the mechanisms behind *T*_2_ is crucial for design of safer lithium-ion batteries.

For the Gr|NMC811 battery with the LiFSI/DMC concentrated electrolyte (Fig. 2a), *T*_2_ was located at 200.5 °C. A fall in the OCV took place at *T*_2_, which coincides with the sharp temperature rise. However, *T*_2_ of the cell with the conventional electrolyte reached 213.1 °C and simultaneously with OCV falling (see Fig. 2b), which is 12.6 °C higher than that of the case with the concentrated electrolyte. *T*_2_ and OCV displayed the repeatable characters (Supplementary Fig. 2), and OCV did not fall until ~213.1/214.8 °C, suggesting that the separators in the cells had the potential to stand 213.1 °C/214.8 °C or even higher temperature without ISC. Additionally, the heat generated by ISC was evaluated based on the internal resistance of battery around *T*_2_. ISC just can contributed a (d*T*/d*t*)_ISC_ of 0.06 °C s^−1^, much lower than 1 °C s^−1^ at *T*_2_ (See details in Supplementary Note 3). Thus, it concludes that, for Gr|NMC811 batteries with the concentrated electrolyte, the exothermic process that leads to *T*_2_ is caused by internal reactions and not by ISC. A large amount of heat was released in the battery. As a consequence, the loss of the integrity of the separator or the battery swell accompanied with the vigorous exothermic reactions, would lead to a sharp drop in the voltage^1,12^. After *T*_2_, the battery temperature sharply increased to the maximum temperature (*T*_3_ = 652.2 °C) in 15.4 s. The maximum d*T*/d*t* during the thermal runaway was 401.2 °C s^−1^. Meanwhile, the violent flame was observed in the lateral heating test (the inset in Fig. 2a), and the overall process is shown in Supplementary Movie 1, indicating that the battery with the concentrated LiFSI/DMC electrolyte was combustible during the thermal runaway even though the electrolyte demonstrated low flammability.

The thermal features of the Gr|NMC532 battery with the concentrated LiFSI/DMC electrolyte were also examined (see Fig. 2c). The chemical reaction was also proved to be the trigger for thermal runaway (see details in Supplementary Note 3). *T*_2_ was found to be 202.3 °C, which is close to that of the Gr|NMC811 battery with the concentrated LiFSI/DMC electrolyte. However, the Gr|NMC532 battery with the conventional electrolyte still showed *T*_2_ at a higher temperature (241.1 °C, see Fig. 2d). It was reported that the crosstalk between the NMC532 cathode and anode was when the reactions happened at *T*_2_^12^. At or after *T*_2_, the most exothermic reactions, where the cathode acted as the main reactant, were initiated. So, it was understandable that Gr|NMC811 batteries always exhibited a lower *T*_2_ than Gr|NMC532 batteries when with the same electrolyte^35^.

It was interesting that the value of *T*_2_ in the Gr|NMC811 battery was very close to that of *T*_2_ in the Gr|NMC532 battery when the same concentrated LiFSI/DMC electrolyte was used in both of them, and both values were lower than that of the *T*_2_ in the battery with the conventional electrolyte. These phenomena indicated that the concentrated LiFSI/DMC electrolyte could not enhance the intrinsic battery safety, even though the concentrated electrolyte was more thermally stable than the conventional electrolyte. Then, the quite similar *T*_2_ of the NMC811 and NMC532 batteries with the LiFSI/DMC concentrated electrolyte indicate that similar chemical reactions occurred at ~200 °C and that these reactions brought both batteries to the point of thermal runaway. To probe the trigger reactions in the batteries, partial cells were employed to simulate all the possible exothermic reactions in the battery.

### Contribution of the exothermic reactions to thermal runaway

**Fig. 3:**
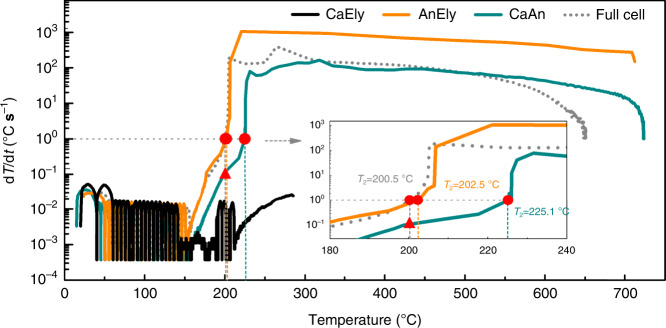
Comparison of thermal runaway features of CaEly, AnEly, and CaAn partial cells with full cell. AnEly, CaEly, and CaAn partial cells were prepared from the fully charged Gr|NMC811 batteries to investigate the contribution of different exothermic reactions during the thermal runaway process of the battery. The temperature dependence of d*T*/d*t* between the full cell (dot line in gray) and the partial cells was compared. No electrolyte in the CaAn cell, while the concentrated LiFSI/DMC electrolyte was used for all the other partial cells and the full battery.

To detect the exothermic reactions of the concentrated LiFSI/DMC electrolyte in Gr|NMC811 battery, a comparison of the temperature dependence of d*T*/d*t* between the full cell and the partial cells is shown in Fig. 3. In contrast to the AnEly and CaAn partial cells, the CaEly partial cell did not enter thermal runaway. No sharp temperature rise was observed from the ARC curve, and its *T*_3_ was 290 °C. Meanwhile, the maximum d*T*/d*t* was even lower than 0.1 °C s^−1^, which is far lower than 1 °C s^−1^. These results illustrate that the heat generated by the reactions inside the CaEly partial cell, including the cathode material decomposition and electrolyte oxidation by the cathode material, was relatively small before 290 °C and incapable of triggering the battery thermal runaway. This result coincides with the low flammability of the concentrated electrolyte, since the combustion reflected the intensity when the electrolyte was oxidized by the oxygen in the air, and the ARC result of the CaEly partial cell reflected the maximum intensity when the electrolyte was oxidized by the charged cathode or the oxygen released by the charged cathode. The AnEly and CaAn partial cells, on the contrary, could be brought to thermal runaway (see Fig. 3). *T*_2_ reached 200.5 °C, 202.5 °C, and 225.1 °C in the case of the full cell, AnEly, and CaAn partial cells, respectively. In addition, the maximum (d*T*/d*t*)_max_ for the full cell, AnEly, and CaAn partial cells was 401.2 °C s^−1^, 882.9 °C s^−1^, and 164.0 °C s^−1^, respectively. Thus, it can be roughly concluded that both the AnEly and CaAn partial cells may contribute to thermal runaway in the full cell, but further analysis is needed to exactly find out which of them provided the trigger reaction and which of them provided the main reaction during thermal runaway.

To analyze the trigger reactions, the d*T*/d*t* values of all the cells at 200.5 °C (*T*_2_ of the full cell) were compared. It was observed that the d*T*/d*t* of the CaAn partial cell was 0.1 °C s^−1^ (see the triangle in Fig. 3) when the d*T*/d*t* of the full cell reached 1 °C s^−1^ (see the circle in Fig. 3), meaning that the heat released by the CaAn partial cell at this temperature was no more than 1/10 of the total heat of the full cell. In fact, the d*T*/d*t* of the CaAn partial cell was always ~1/10 of that of the full cell. Since the decomposition of the cathode material released little heat, the reactions inside the CaAn partial cell contributed little to *T*_2_ and the heat accumulation before *T*_2_ for the full cell. As a result, the CaAn partial cell cannot provide the trigger reaction. In contrast, the d*T*/d*t* of the AnEly partial cell was close to 1 °C s^−1^ at *T*_2_, and the d*T*/d*t* curves of the AnEly and full cell almost overlapped near *T*_2_. This demonstrates that the heat release dynamics of the AnEly and full cell were almost the same. Thus, the chemical reactions between the anode-electrolyte were exactly the same reactions that took place in the full cell. Since the battery was uniformly heated and the cell was so small that its thermal conductivity was quite good, the reactions would uniformly take place inside the cell. In addition, the exponential growth of the d*T*/d*t* with the temperature would be attributed to exothermic chemical reactions. That is, multiple reactions were involved between the anode and the electrolyte, and they were also initiated one after the other with a rise in temperature. In turn, the released heat led to a continuous increase in the temperature of the full cell. When the cell temperature became close to *T*_2_, a group of vigorous reactions was initiated, enabling the full cell temperature surge at *T*_2_. This chain reaction is very complex, and its analysis would be a great chance for future research. Overall, the rise in the temperature curves of the AnEly and full cells was very similar below *T*_2_, which proves that the AnEly cell was responsible for the trigger reaction that led to the battery’s thermal runaway.

The surge after *T*_2_ could be obviously observed in the full, AnEly, and CaAn cells. Then, the d*T*/d*t* gradually decreased until the cell temperature reached the maximum value. Although the maximum temperature (*T*_3_) of the three kinds of cells is different, the maximum d*T*/d*t* of the AnEly and CaAn cell was both brought to hundreds of orders of magnitude, which indicates that the reactions in both the AnEly and CaAn cells were mainly responsible for the exothermic reactions during thermal runaway. Also, *T*_3_ of each of the AnEly and CaAn cells was higher than that of the full cell because some of the battery components did not exist in the AnEly and CaAn partial cells.

*Q*_TR_ was used to denote the intensive heat release during thermal runaway, and it can be calculated from Eq. (1)^13^, where *M* denotes the mass of the cell (g), and *C*_p_ signifies the specific heat capacity (J·g^−1^ K^−1^; Supplementary Table 2 and Supplementary Note 4). The temperature range for the heat calculation is Δ*T* (Δ*T* = *T*_3_−*T*_1_). Table 1 shows the characteristic temperatures and *Q*_TR_ of the cells, and the equivalent rise in temperature of full battery (Δ*T*_eq_) which was caused by the reactions in the partial cells. Δ*T*_eq_ was calculated from Eq. (2). The AnEly partial cell released a *Q*_TR_ of 7.0 kJ with a Δ*T*_eq_ of 312.6 °C, whereas the full cell released a *Q*_TR_ of 11.5 kJ with a Δ*T* of 516.9 °C. Meanwhile, the CaAn partial cell generated a *Q*_TR_ of 11.3 kJ with a *ΔT*_eq_ of 507.9 °C, implying that the redox reactions between the anode–cathode can also generate tremendous heat from *T*_2_ to *T*_3_^12,13^. The AnEly partial cell, among the three partial cells, was first brought to a thermal runaway. If the AnEly partial cell contributed all the heat to the full cell, the reaction in the CaAn cell would at least provide heat of 4.5 kJ to the full cell. It indicates that the reactions in both the AnEly and CaAn cells were the main reactions during the thermal runaway. Besides, even if the cathode was completely inert under all the temperatures, the heat released by the reactions between the anode and the electrolyte can bring the full battery to thermal runaway state (see details in Supplementary Table 3 and Supplementary Note 5). Similarly, the full cell can be brought to thermal runaway but at a higher temperature if the electrolyte stays totally inert.

1 $$Q_{{\mathrm{TR}}} = {\mathrm{{\Delta}}}T{\sum} ({M\cdot C_{\mathrm{p}}})$$

2 $${\mathrm{{\Delta}}}T_{{\mathrm{eq}}} = \frac{{Q_{{\mathrm{TR}}}}}{{{\sum} ({M\cdot C_{\mathrm{p}}}) }}$$

**Table 1:** Thermal runaway features of the partial cells and full cell.

Cells	Mass (g)	*T*_1_ (°C)	*T*_2_ (°C)	*T*_3_ (°C)	Δ*T* (°C)	(d*T*/d*t*)_max_ (°C S^−1^)	*Q*_TR_ (kJ)	Δ*T*_eq_ (°C)
CaEly	13.9	220.3	–	288.3	68	–	0.8	37.4
AnEly	13.9	130.1	202.5	712.2	582.1	882.9	7.0	312.6
CaAn	17.5	140.7	225.1	724	583.3	164	11.3	507.9
Full cell	24.5	135.3	200.5	652.2	516.9	401.2	11.5	–

Based on the above analysis, three conclusions can be drawn for the Gr|NMC811 thermal runaway. First, the reactions between the cathode–anode contributed little to *T*_2_ and the heat accumulation before *T*_2_, which coincides with the low flammability of the concentrated electrolyte. Second, the reactions in the AnEly partial cell were responsible for the heat accumulation below *T*_2_, as well as the trigger reaction of the thermal runaway. Third, the reactions in both the AnEly and CaAn partial cells were the main reactions during the thermal runaway. In the following section, the thermal stabilities of the individual materials and their mixtures are estimated to further probe the trigger reactions and main exothermic reactions during thermal runaway.

### Thermal stability of LiFSI/DMC in Gr|NMC811 battery

**Fig. 4:**
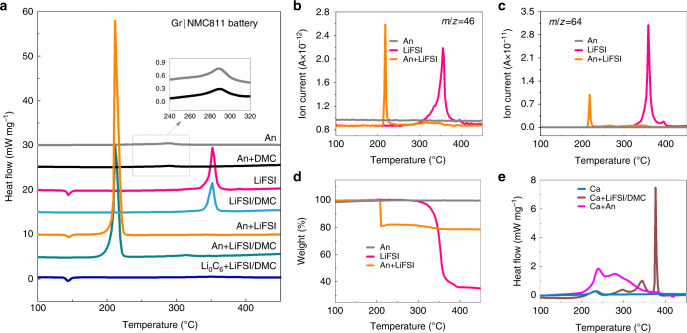
Thermal stability of cell components and their mixtures in the Gr|NMC811 battery. **a** DSC traces of the lithiated anode, concentrated LiFSI/DMC electrolyte components, and their mixtures for the Gr|NMC811 battery. The inset displays the enlarged peaks of An and An + DMC. **b** NO_2_ (*m*/*z* = 46) gas evolution of LiFSI, the lithiated anode and their mixture during the DSC measurement. **c** SO_2_ (*m*/*z* = 64) gas evolution of LiFSI, the lithiated anode, and their mixture during the DSC measurement. **d** The weight loss of the lithiated anode, LiFSI, and their mixture. **e** DSC traces of the cathode, cathode mixed with concentrated LiFSI/DMC, and cathode mixed with anode.

A DSC-TG-MS test was used to characterize the thermal stability of the cell components. By enumerating all the thermal reactions of the individual and mixed cell components, the reactions inside the battery during the thermal runaway evolution can be screened out. As the chemical reactions in the AnEly partial cell were targeted as the trigger reaction of thermal runaway. Then, first, all the possible reactions among the lithiated anode, concentrated LiFSI/DMC electrolyte, electrolyte components, and delithiated cathode were measured (Fig. 4 and Supplementary Table 4).

Both the lithiated anode (An) and the An+DMC mixture exhibited a broad and mild exothermic peak at ~289 °C (the dotted box and the inset in Fig. 4a), where the corresponding Δ*H* for both of them was ~70 J g^−1^. This exothermic peak can be attributed to the reaction between the lithiated graphite and the polyvinylidene fluoride-based binder^12^, which coincides with what was indicated by the plat TGA curve (Fig. 4d). In detail, the weight was kept constant in the temperature range between the room temperature and 550 °C, which is consistent with the fact that no gases or volatilizable liquids were produced in the reactions between the lithiated graphite and the binder. The addition of DMC did not change it, indicating that no reaction between the An and DMC occurred. For LiFSI, the endothermic peak at ~145 °C can be attributed to the melting of LiFSI, as no weight loss at this temperature occurred (Fig. 4d). As for the concentrated electrolyte, the peak at ~145 °C disappears (LiFSI/DMC and An+LiFSI/DMC curves in Fig. 4a). The exothermic peak at ~350 °C might be associated with the thermal decomposition of FSI^–^^36,37^, as 65% of the weight loss can be determined according to Fig. 4d, and the NO_2_ (*m*/*z* = 46) and SO_2_ (*m*/*z* = 64) gases were released at ~350 °C because of the S–F and S–N breakage in the FSI^−^^36,37^ (Fig. 4b, c). The concentrated electrolyte and LiFSI showed quite a similar exothermic peak at ~350 °C, which indicates the high thermal stability between LiFSI and DMC. Besides, the change from the crystal to the solution state did not change the decomposition behavior of LiFSI.

However, the addition of the lithiated anode results in obvious changes in the thermal behavior of LiFSI, which signifies the reaction between them. As seen in Fig. 4a, for the mixture of LiFSI and An_,_ when the lithiated anode was in contact with the concentrated LiFSI/DMC electrolyte, the sample showed a sharp exothermic peak (602.9 J g^−1^) at 209.6 °C, which was eight times greater than that of the anode alone, while no exothermic peaks took place at 350 °C. Similarly, for the An+LiFSI, NO_2_ and SO_2_ gases were released at 210.9 °C, and they were accompanied by an intensive Δ*H* of 757.9 J g^−1^. In addition, the TGA curve (Fig. 4d) showed that a weight loss of almost 20% took place at ~210 °C, while the weight loss at ~350 °C was <3%, indicating that most of the LiFSI powders reacted with the lithiated anode and produced gas or volatile products. It is reasonable that the DSC curve of the An+LiFSI/DMC sample did not show a LiFSI decomposition, as the heat released by the 3% residue of the LiFSI was too little. Furthermore, for the fresh graphite (Li_0_C_6_), Li_0_C_6_ + LiFSI displayed an endothermic peak at 145 °C without the intensive exothermic behavior at ~210 °C, demonstrating the considerable heat produced in LiC_6_ + LiFSI from the chemical reaction between intercalated lithium and LiFSI. It was also reported in ref. ^38^ that the battery employing the LiFSI-based electrolyte showed an exothermic peak of 1300 J g^−1^ at ~200 °C, attributed to the chemical reduction of the FSI^−^ anion by the lithiated anode^38^. For comparison, the thermal behaviors of LiPF_6_ and An+LiPF_6_ were also investigated, it demonstrated that LiPF_6_ was not involved in the trigger or main reactions of thermal runaway, coincided with the different *T*_2_ values of the concentrated and conventional electrolytes (see details in Supplementary Fig. 3 and Supplementary Note 7).

As shown in Fig. 4e, the NMC811 cathode at the full charge state (Ca) presented a small Δ*H* of 100.6 J g^−1^ at 235.1 °C, which can be attributed to the phase transition. The thermal behavior of Ca+LiFSI/DMC implies that the NMC811 cathode barely reacts with the concentrated LiFSI/DMC electrolyte before 320 °C (see details in Supplementary Note 6). The exothermal peak at 350 °C and 380 °C in Ca+LiFSI/DMC may not be triggered in CaEly partial cell as ARC system will enter into a cooling mode if the cell does not go to thermal runaway at a pre-set temperature of 290 °C. Thus, the exothermic reaction after 320 °C contributed less to the thermal runaway which coincides with the thermal runaway behavior of the CaEly partial cell. Additionally, Ca+Separator was also investigated and with a small heat generation (Supplementary Fig. 4 and Supplementary Note 8). The Ca+An sample exhibited two major exothermic peaks that were centered at 239.5 °C and 279.4 °C, respectively. The Δ*H* values of the two exothermic reactions were calculated, and they were found to be 834.0 J g^−1^ in total, where it was believed that they emanated from the consumption of the cathode-produced oxygen by the anode^12^. The reaction between the cathode and the anode also significantly contributed to the heat during thermal runaway, but it was not the trigger reaction, which coincides with the *Q*_TR_ analysis and thermal runaway behavior of the CaAn partial cell.

Two conclusions can be drawn from Fig. 4. First, the LiFSI can be reduced by the charged anode with gas evolution and the released intensive heat at ~210 °C, which was the trigger reaction that brought the battery to the point of thermal runaway. Second, the reaction between the fully charged cathode and the anode generated great heat, which significantly contributed to the released heat during thermal runaway, but it was not the trigger reaction.

### Thermal stability of LiFSI/DMC in Gr|NMC532 battery

**Fig. 5:**
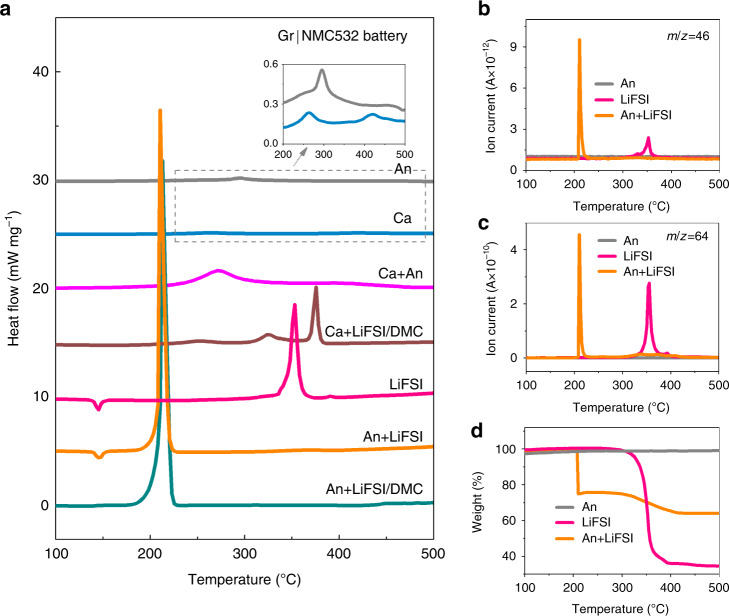
Thermal stability of cell components and their mixtures in the Gr|NMC532 battery. **a** DSC traces of the lithiated anode, cathode, concentrated LiFSI/DMC electrolyte components, and their mixtures for the Gr|NMC532 battery. The inset displays the enlarged peaks of Ca and An. **b** NO_2_ (*m*/*z* = 46) gas evolution of the lithiated anode, LiFSI, and their mixture during the DSC measurement. **c** SO_2_ (*m*/*z* = 64) gas evolution of the lithiated anode, LiFSI, and their mixture during the DSC measurement. **d** The weight loss of the lithiated anode, LiFSI, and their mixture.

It is widely accepted that charged cathodes take part in the thermal runaway process and that the NMC532 battery is more thermally and chemically stable than the NMC811 battery when the other battery materials are the same. According to research on the NMC811 battery with the LiFSI/DMC concentrated electrolyte, the charged cathode only participated in the thermal runaway, while the trigger reaction was led by the anode and the concentrated electrolyte. Also, the Gr|NMC532 battery was further used to confirm thermal runaway evolution when employing concentrated LiFSI/DMC electrolytes (Fig. 5). Similar to the case of the Gr|NMC811 battery, the An and Ca individual samples resulted in a very weak exothermic peak, with Δ*H* values of 68.2 and 46.8 J g^−1^, respectively (the insert in Fig. 5a and Supplementary Table 5), indicating that without a strong oxidizer or redactor, An and Ca cannot cause great damage by thermal decomposition. As for their mixtures, two peaks appeared at 272.1 °C and 394.3 °C with a total Δ*H* of 709.3 J g^−1^. This intensive heat was considered to bring the Gr|NMC532 battery with the conventional electrolyte to thermal runaway^12^. However, for the concentrated electrolyte, the LiFSI exhibited an energetic peak of ~350 °C (Fig. 5a). In contrast, the An+LiFSI mixture only showed one exothermic peak at 210.5 °C with a Δ*H* of 767.8 J g^−1^ and with a simultaneous release of NO_2_ and SO_2_ gases (see Fig. 5a–c). This change indicates that reactions took place between An and LiFSI. Similar to the results in the Gr|NMC811 battery, where the An induced the damage of the S–F and S–N bonds in the LiFSI with an intensive heat generation of ~210 °C, the heat was considerably high to cause thermal runaway. Figure 5d shows the TGA curves of the lithiated anode and LiFSI and their mixture. The DSC-TG-MS characteristics of the lithiated anode, electrolyte components, and their mixtures in the Gr|NMC532 battery coincided with the case of the Gr|NMC811 battery. Herein, for Gr|NMC532 battery with the concentrated LiFSI/DMC, the trigger reaction was also demonstrated to be LiC_6_ + LiFSI.

### Non-flammable LiFSI/TMP in Gr|NMC811 battery

**Fig. 6:**
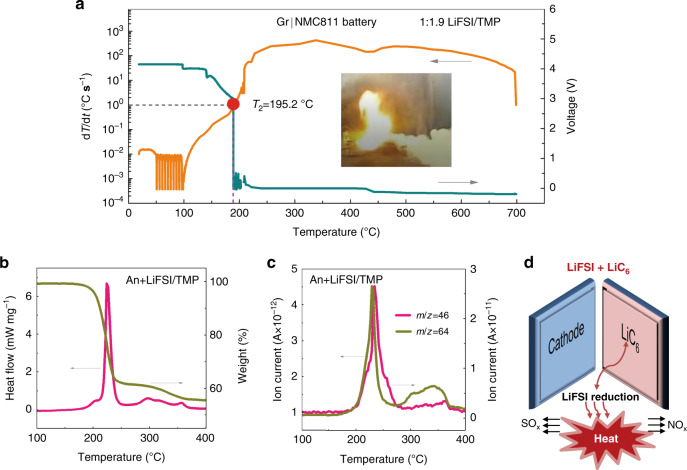
Thermal runaway of Gr|NMC811 battery with non-flammable concentrated LiFSI/TMP. **a** The temperature dependence of d*T*/d*t* of the Gr|NMC811 battery with the concentrated LiFSI/TMP. The inset shows the combustibility of battery in the lateral heating test. **b** DSC trace and TGA curve of the An+LiFSI/TMP sample. **c** NO_2_(*m*/*z* = 46) and SO_2_(*m*/*z* = 64) gas evolution of the An+LiFSI/TMP sample. **d** Illustration of the proposed thermal runaway mechanism of LiFSI-based concentrated electrolytes in Gr|NMC batteries. The considerable heat generated by the reaction of LiFSI+LiC_6_ triggers the Gr|NMC batteries to thermal runaway.

The release of DMC below *T*_2_ was an interference factor when analyzing the combustion of the Gr|NMC battery with the LiFSI/DMC concentrated electrolyte, as the small amount of free DMC is flammable though the flammability is greatly reduced. Then, the non-flammable concentrated LiFSI/TMP electrolyte was also examined in the Gr|NMC811 battery to further verify the vigorous reactions between the lithiated graphite and the LiFSI. Unfortunately, the battery was brought to thermal runaway at a *T*_2_ of 195.2 °C (Fig. 6a), which is even less than the *T*_2_ of the battery with the LiFSI/DMC concentrated electrolyte. As shown in Fig. 6b, c, the An+LiFSI/TMP sample showed an intensive exothermic peak at ~210 °C, which was accompanied by a release of NO_2_ and SO_2_ gases. The Δ*H* was found to be 540.4 J g^−1^, which is lower than that of the LiFSI/DMC concentrated electrolyte. However, the TMP could not prevent the exothermic reactions of LiC_6_ + LiFSI. As a result, the thermal runaway of the battery can still be triggered and then proceeded even with the non-flammable electrolyte. Meanwhile, a violent flame could still be observed in the lateral heating test (the inset in Fig. 6a and Supplementary Movie 2). This indicates that, although the concentrated LiFSI/TMP electrolyte was non-flammable, the reactions between the anode and the electrolyte, as well as the reactions between the cathode and the anode, were vigorous enough to cause a strong fire. It is known that combustion takes place as a result of the reaction between the electrolyte and oxygen. Herein, for LiFSI-based concentrated electrolytes, the trigger reaction was between the anode and the electrolyte (Fig. 6d), and the reaction that contributed to the thermal runaway was the redox reaction between the cathode–anode. Both reactions had nothing to do with the flammability of the electrolytes. Thus, the battery safety cannot be estimated based on the flammability of the used electrolytes. Overall, the complex reactions among the cell components should be carefully taken into consideration for battery safety assessments.

### Post-test analysis

An XPS analysis was conducted on the DSC residue to further support the thermal runaway mechanism. During the DSC measurement, the reaction of the LiFSI with the lithiated anode was ceased at 230 °C, which was at the end of the exothermic peak (Supplementary Fig. 5a). After cooling to room temperature, the sample was transferred for the XPS analysis (Supplementary Fig. 5b–e). The byproducts validated the chemical reactions between the LiFSI and LiC_6_ during the thermal runaway, supposing that the tremendous heat was initiated by breaking the S-F and S-N bonds with the formation of Li_2_CO_3_, Li_2_SO_3_, Li_2_SO_4_, LiF, and so on (see details in Supplementary Note 9). The XPS analysis of An+LiFSI/TMP was also investigated (Supplementary Fig. 6 and Supplementary Note 10).

## Discussion

This study revealed that batteries with LiFSI-based concentrated electrolytes also undergo thermal runaway. In the conducted experiments in this study, although the fire-retardant agent acted as a solvent, lithium salt acted as a strong oxidant, and the fire-retardant agent could not hinder the reactions between the lithium salt and the lithiated anode. Also, the reactions between the anode and the cathode were found to significantly contribute to the heat output during thermal runaway, where the fire-retardant agent could not work. The flammability of electrolytes does contribute to thermal runaway for the batteries with conventional flammable electrolytes, but it is not the only contributable reaction and it is even not the biggest contributor to the strong cathode oxidization like the NMC811. Thus, the interactions between the charged electrodes and electrolytes should be fully considered in battery safety assessments. These findings provide valuable insights into the thermal runaway mechanisms of concentrated electrolytes, including water-in-salt aqueous electrolytes in LIBs.

## Methods

### Materials and batteries

The solvents of EC (ethylene carbonate) and EMC (ethyl methyl carbonate) and the electrolytes of LiFSI/DMC (1:1.9 by molar), LiFSI/TMP (1:1.9 by molar), and 1 M LiPF_6_ in EC:EMC (3:7 by volume) were purchased from Dodochem Ltd. The moisture content of the solvents and electrolytes was <20 ppm. 0.95 Ah Gr|NMC811 and 1.2 Ah Gr|NMC53 pouch cells with a ceramic coated PE separator were used in this work. The Gr|NMC811 and Gr|NMC532 dry cells were manufactured by Guangdong Canrd New Energy Technology Co., Ltd. The LiFSI/DMC and LiFSI/TMP concentrated electrolytes were injected into the dry cells with 3.6 mL per Ah, and the formation of all the batteries was performed at C/10 under 45 °C. The cells were cycled in the voltage range of 2.85–4.2 V at 1/3 C under 25 °C. The energy densities of the Gr|NMC811 and Gr|NMC532 batteries were 191 Wh kg^−1^ and 182 Wh kg^−1^, respectively (see details in Supplementary Table 6 and Supplementary Note 11). All the batteries were charged to 4.2 V before the disassembly and measurements.

### Battery safety evaluation

**Fig. 7:**
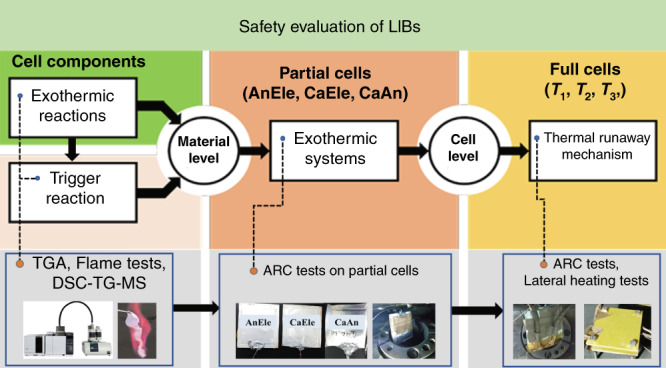
Safety evaluation of LIBs. Characterization of battery safety at the cell and material levels and the corresponding tests.

Battery safety was systematically assessed at the cell and material levels in this study as shown in Fig. 7. In detail, the thermal properties of the concentrated electrolytes were characterized by an ignition test, TGA and DSC. The safety performance of the battery was evaluated by an ARC test. The combustibility of the battery was measured by lateral heating. DSC-TG-MS was used to probe the reactions during the thermal runaway evolution, revealing the thermal runaway mechanism. Partial cells were used to simulate the reactions between different battery components in the battery environment, and an ARC test was employed to record the heat flow. The reactions between the different battery components were also investigated with DSC-TG-MS, and the results provided the reaction dynamics in the material level.

The assembly of the partial cells is shown in Supplementary Fig. 7 and Supplementary Note 12, and the detailed description is presented in ref. ^11^. After removing the cathode from the fully charged Gr|NMC811 battery, the cell that only retains the lithiated anode and concentrated LiFSI/DMC was sealed and named as the AnEly partial cell. Similarly, the CaEly partial cell was prepared. As for the CaAn partial cell, the charged cathode and anode were washed with DMC and naturally dried, and then the cathode and anode were rolled without a separator to assemble the CaAn partial cell^33^. All the above processing steps were performed in an argon-filled glove box in which the oxygen and water contents were controlled below 0.1 ppm. The mass of active materials in AnEly, CaEly, and CaAn were exactly the same with the full cell, and these three kinds of partial cells represent the main thermodynamic systems in the battery. The ARC test was then applied to measure these partial cells so as to analyze the trigger reaction of the thermal runaway.

### ARC test protocol

A standard accelerated rate calorimetry (ARC-ES) instrument with an internal diameter of 10 cm and a depth of 10 cm, which was manufactured by Thermal Hazard Technology, was used to evaluate the thermal runaway features. The heat-wait-seek method was conducted on the ARC to detect the adiabatic self-heating rate as a function of time^1,13^. A heating step of 5 °C with a wait time of 15 min was performed on the ARC starting from 40 °C. The characteristic temperatures (*T*_1_, *T*_2_, and *T*_3_) were extracted based on the data analysis. *T*_1_ is defined as the self-heating temperature of the battery, where self-heating is identified when the battery’s temperature increase rate reaches 0.02 °C min^−1^ without external heat. *T*_2_ refers to the onset temperature of the thermal runaway, which is recorded when the battery’s temperature rise rate (d*T*/d*t*) reaches 1 °C s^−1^. *T*_3_ is the battery’s highest temperature during the thermal runaway^1,13^. Note that ARC system will enter into the cooling mode if the cell does not go to thermal runaway at a pre-set temperature of 290 °C. Meanwhile, the real-time open-circuit voltage (OCV) of the battery was recorded during the ARC test.

Before the ARC test, micro-thermocouples were inserted into the internal central positions of the tested partial and full cells as shown by the red arrows (Supplementary Fig. 7a). Then, the cells were resealed with a silicone sealant that can maintain tightness and elasticity even at 343 °C. The micro-thermocouples probed the real-time temperature of the cells during the ARC measurement.

### Lateral heating test protocol

The combustibility of the Gr|NMC811 batteries with LiFSI/DMC (1:1.9 by molar) and LiFSI/TMP (1:1.9 by molar), respectively, was examined by a lateral heating test^39^. In detail, a ceramic heater, 28 mm in diameter, was fixed on the center of the battery’s surface. Then, the battery and the pad were clamped together with two epoxy plates (Supplementary Fig. 7b). When the measurement started, the ceramic heater was connected with a 20 W DC power to heat the battery to 200 °C. The whole process was video-recorded, and the combustibility feature of the battery was analyzed.

### DSC-TG-MS test protocol

A DSC-TG-MS test was used to characterize the thermal properties and gaseous products of the cell components. The experiments were performed on NETZSCH STA449F5-QMS403D. The anode and cathode samples were acquired by disassembling a fully charged battery. The electrode was firstly washed with DMC to remove the adsorbed electrolyte, and then the cathode and anode powders were scratched from the electrodes in an argon-filled glove box and then dried at 60 °C in the same glove box. All the samples were pressed in an aluminum crucible in the glove box and heated from 50 °C to 600 °C at a heating rate of 10 °C min^−1^ in a highly pure flowing argon atmosphere. The examined individual cell components or their mixtures by the DSC-TG-MS test are listed in Table 2. These eleven samples covered the key exothermic reactions in the battery^33^. In the DSC results, the heat flow was normalized by the total weight of Ca+An+LiFSI/DMC, which was calculated based on the measured weight of the test sample and the corresponding weight ratio of the test sample in Ca+An+LiFSI/DMC. The composing proportion of Ca+An+LiFSI/DMC is the same as that of the full cell. By these means, the peak intensity implies the heating effect on the battery. The heat generations (Δ*H*) normalized by both the individual component weight and total weight are shown in Supplementary Tables 4 and 5.

**Table 2:** Samples for DSC-TG-MS test.

Sample no.	Composition	Mass (mg)	Scanning rate (°C min^−1^)
1	Cathode (Ca)	4.0	10
2	Anode (An)	3.4	10
3	LiFSI/DMC	4.6	10
4	An+LiFSI/DMC	3.4 + 4.6	10
5	An+DMC	3.4 + 2.2	10
6	An+LiFSI	3.4 + 2.4	10
7	Li_0_C_6_+LiFSI	3.4 + 2.4	10
8	Ca+LiFSI/DMC	4.0 + 4.6	10
9	Ca+DMC	4.0 + 2.2	10
10	Ca+LiFSI	4.0 + 2.4	10
11	Ca+An	4.0 + 3.4	10

### XPS analysis

To probe the byproducts of the reactions between the lithiated anode and the concentrated electrolyte, the sample for XPS (X-ray photoelectron spectroscopy) analysis was prepared as follows. First, a mixture of the full charged anode and certain amounts of the electrolyte was heated to 230 °C with a heating rate of 10 °C min^−1^. Then, the solid residues were cooled to room temperature in flowing argon, collected, and analyzed by XPS, which was collected with a K-Alpha + spectrometer using the Thermo Fisher Scientific Co. The binding energy (BE) scale was calibrated with a C 1 s peak at 284.8 eV.

## Supplementary information

Supplementary Information

Description of Additional Supplementary Files

Supplementary Movie 1

Supplementary Movie 2

## Data Availability

The data that support the findings of this study are available from the corresponding author upon reasonable request.
