# Supplementary material for: Thermal runaway of Lithium-ion batteries employing LiN(SO2F)2-based concentrated electrolytes
Source: Nat Commun. 2020 Oct 9;11:5100. doi: 10.1038/s41467-020-18868-w (PMC7547674; doi:10.1038/s41467-020-18868-w)
Supplement: Supplementary file 1 — Supplementary Information [file 41467_2020_18868_MOESM1_ESM.pdf]

SUPPLEMENTARY INFORMATION

Thermal runaway of Lithium-ion batteries employing $LiN(SO_2F)_2$-based concentrated electrolytes

Junxian Hou[a], Languang Lu[a], Li Wang[b]*, Atsushi Ohma[c], Dongsheng Ren[a], Xuning Feng[a], Yan Li[a], Yalun Li[a], Issei Ootani[c], Xuebing Han[a], Weining Ren[a], Xiangming He[b], Yoshiaki Nitta[c], Minggao Ouyang[a]*

[a]State Key Laboratory of Automotive Safety and Energy, Tsinghua University, Beijing 100084, China

[b]Institute of Nuclear and New Energy Technology, Tsinghua University, Beijing 100084, China

[c]Advanced Materials and Processing Laboratory, Nissan Motor Co., Ltd., 1, Natsushima-cho, Yokosuka, 237-8523, Japan

*E-mail: wang-l@mail.tsinghua.edu.cn; ouymg@mail.tsinghua.edu.cn

Supplementary Note 1: Charge and discharge performance of Gr|NMC532 batteries

The charge and discharge performance of Gr|NMC532 batteries with concentrated LiFSI/DMC, and conventional electrolytes were examined for two cycles before the safety evaluation. In detail, the concentrated LiFSI/DMC and the conventional electrolyte in Gr|NMC532 batteries both displayed a reversible capacity of 1.23 Ah for the second cycle and a coulombic efficiency of 99.7% and 99.9%, respectively (Supplementary Fig. 1). After operating for two cycles, the batteries were fully charged to 4.2V for the ARC tests and the material analysis.

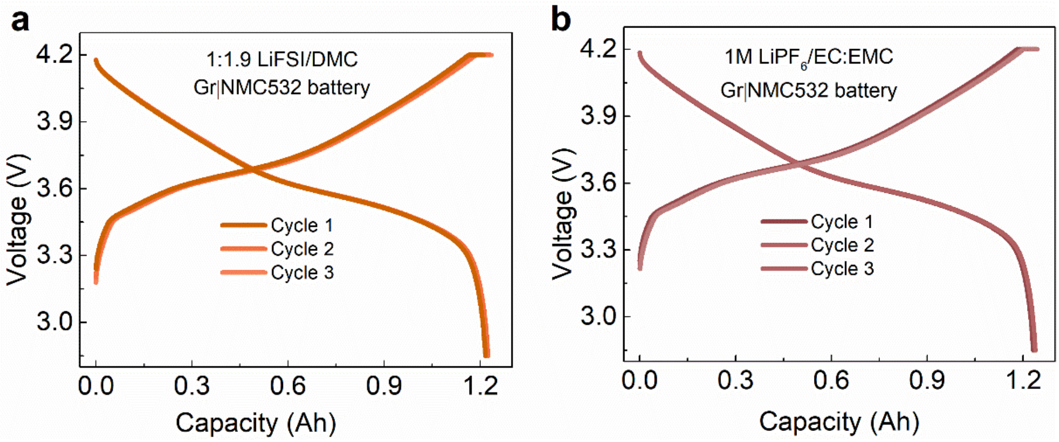

Supplementary Fig. 1 a Charge and discharge plots of the Gr|NMC532 battery with the concentrated LiFSI/DMC (1:1.9 by molar). **b** Charge and discharge plots of the Gr|NMC532 battery with the conventional electrolyte (1M $LiPF_6$ in EC:EMC).

Supplementary Note 2: Flash points of the solvents

Flash point can be used to illustrate the ease of material to be ignited. In this study, the solvents of EC, EMC, DMC were reported to show the flash points of 145.5 ± 4℃, 23.5 ± 1℃, 16 ± 1℃ respectively, and TMP did not show flash point up to 150℃[1], as shown in Supplementary Table 1. For the electrolyte, 1M $LiPF_6$/EC:EMC (3:7 vol) is highly volatile and flammable and can be readily ignited at around room temperature, and was reported to show the flash point of 28 ± 1℃[1]. The concentrated LiFSI/TMP was reported to show no valid flash point up to their boiling points (> 200 °C)[2].

Supplementary Table 1. Flash points of the solvents[1].

Substance	EC	EMC	DMC	TMP
Flash point (℃)	145.5 ± 4	23.5 ± 1	16 ± 1	No flash point< 150

Supplementary Note 3: Repetition of ARC tests and heat contribution of ISC at T_2

As shown in Supplementary Fig. 2, in the repetition of ARC tests, T_2 and OCV displayed the repeatable characters. In specific, T_2 of NMC811 – LiFSI/DMC and NMC532 – LiFSI/DMC showed very similar values, which were 202.6℃ and 203.7℃ respectively, and a fall in the OCV took place right at T_2. For NMC811 – $LiPF_6$/EC:EMC case, T_2 displayed a close value (214.8℃) and also OCV fell simultaneously. These repeatable experimental results demonstrated that the falling of OCV was caused by the intrinsic characters of batteries rather than the separator's random errors. Additionally, NMC532- $LiPF_6$/EC:EMC cell showed higher T_2 than NMC811 – $LiPF_6$/EC:EMC cell was observed already[3], confirming the strong correlation between OCV drop and intense reactions.

To demonstrate the role of ISC during thermal runaway, the internal resistance of battery was detected at a constant AC frequency of 1 kHz with an external battery resistance tester during the entire ARC test for the LiFSI/DMC cases. Plots of the temperature rate, cell voltage, and resistance versus the temperature are shown in Supplementary Figs. 2a and b. It can be found that, for NMC811 – LiFSI/DMC case, the internal resistance of the battery slowly increased from 42 mΩ at room temperature to around 2900 mΩ before and at T_2. The huge internal resistance increasement during thermal runaway was also reported in the literatures 3 and 4[3, 4]. The reason would be gas production and/or the depletion of electrolyte. Normally, during ARC test, the battery was staying under high temperature (for example, T_{cell} > 100 ℃) more than 10 hours before thermal runaway (Supplementary Fig. 2d), leading to intensive volatilization of electrolyte. Thus, lithium ion transportation was blocked and huge resistance increase would be observed. The joule heat produced by internal resistance around T_2 with a voltage of 3.90 V can be calculated based on the internal resistance according to Supplementary Equation 1. The temperature rise rate caused by ISC [$(dT/dt)_{ISC}$] can be calculated by Supplementary Equation 2. The efficiency factor (ξ) in Supplementary Equations 1 and 2 is a parameter defined to represent the fraction of the maximum stored electrical energy that converts into thermal energy, as illustrated in Ref. 5 – 7[5-7]. Based on Supplementary Equations 1 and 2, $(dT/dt)_{ISC}$ is calculated with a value of 0.06 ℃ s^{-1}, much lower than the real dT/dt of battery with 1 ℃ s^{-1} at T_2. It proves that ISC has a small contribution to thermal runaway, cannot be the trigger of thermal runaway.

$$Q_{\mathrm{ISC}} = \xi \cdot \frac{U^2}{R} \quad \text{(Supplementary Equation 1)}$$

$$(\mathrm{d}T/\mathrm{d}t)_{\mathrm{ISC}} = \frac{Q_{\mathrm{ISC}}}{M \cdot C_{\mathrm{p}}} = \xi \cdot \frac{U^2}{R \cdot M \cdot C_{\mathrm{p}}} \quad \text{(Supplementary Equation 2)}$$

$$(\mathrm{d}T/\mathrm{d}t)_{\mathrm{ISC}} = 0.06 \text{ ℃ s}^{-1}$$

Where Q_{ISC} is the heat generation power of ISC, $(dT/dt)_{ISC}$ is the temperature rise rate caused by ISC. U is the OCV of the battery as shown in Supplementary Fig. 2a, ζ was calculated with a value of 0.28 in the Ref. 5 for a single cell and it also considers the fraction of energy that leaves with the gases and the solids ejected during thermal runaway[5]. R is the internal resistance of battery during ARC test as shown in Supplementary Fig. 2. M is the battery mass with a value of 24.5 g, as shown in Table. 1.

Similarly, the internal resistance of NMC532 – LiFSI/DMC battery was also recorded during ARC test (Supplementary Fig. 2b). The internal resistance increased from 40 mΩ at room temperature to 2300 mΩ around T_2. It is calculated that $(dT/dt)_{ISC}$ has a value of 0.05 ℃ s^{-1}, also much lower than that the real battery's dT/dt of battery with 1 ℃ s^{-1} at T_2. This also proves that ISC has a small contribution to thermal runaway, cannot be the trigger of thermal runaway for NMC532 – LiFSI/DMC case.

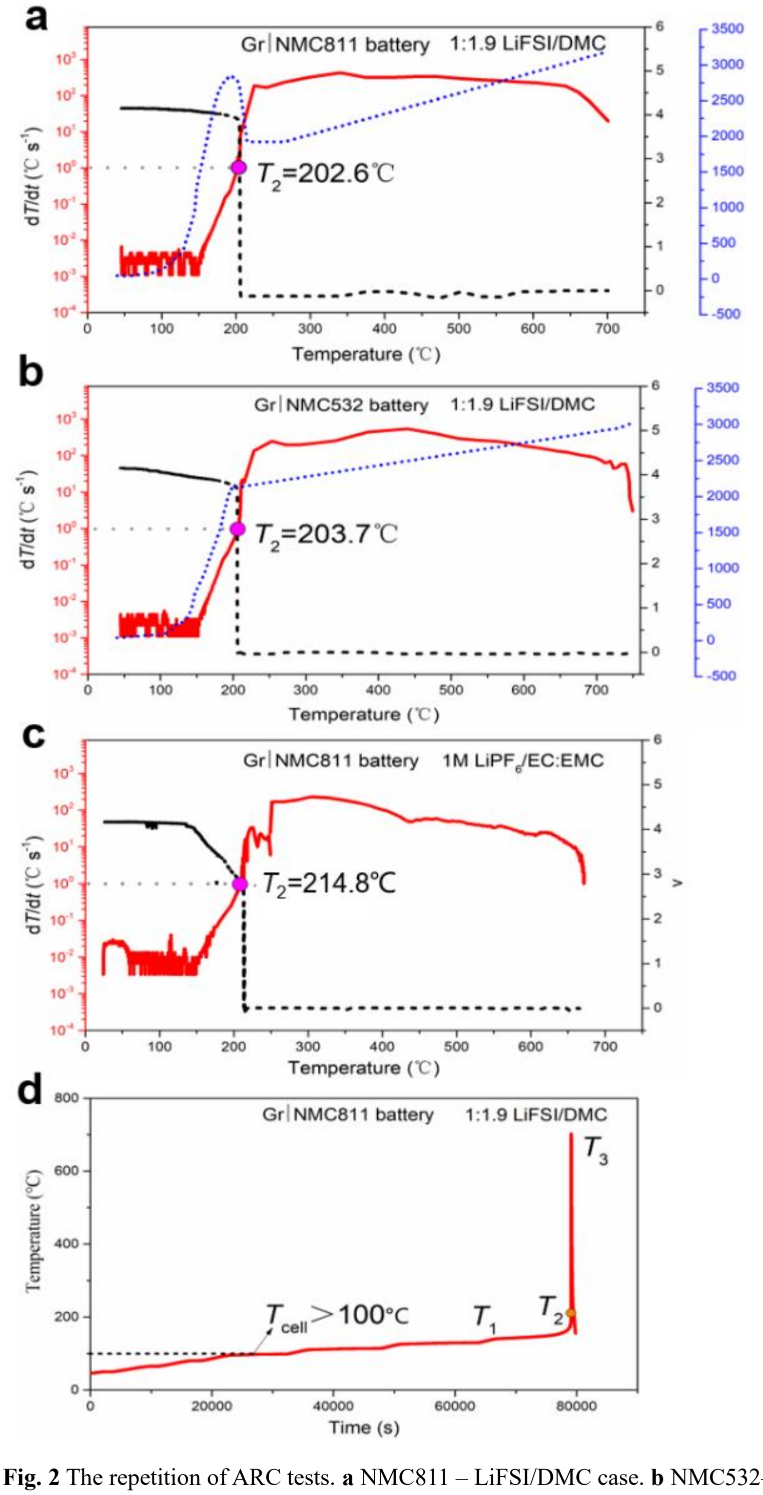

Supplementary Fig. 2 The repetition of ARC tests. **a** NMC811 – LiFSI/DMC case. **b** NMC532– LiFSI/DMC case. **c** NMC811 – $LiPF_6$/EC:EMC case. **d** Time dependence of temperature curve of NMC811 – LiFSI/DMC case during the ARC test.

Supplementary Note 4: Details of the Gr|NMC811 battery

Supplementary Table 2 displays the details of the Gr|NMC811 battery, including the cell components, mass (*M*), and heat capacity (C_p). For the cells, the heat cannot be calculated accurately from ARC data using equation (1), as equation (1) fails to take into account the heat losses that take place in the ARC experiments where high temperature rates are attained.

Supplementary Table 2. Details of the Gr|NMC811 battery, including the cell components, mass (*M*), and heat capacity (C_p).

Component	Material	*M*(g)	C_p [J kg^{-1}K^{-1}]
Cathode	NMC811	5.2	1270[11]
Anode	Graphite	4.42	1437[11]
Separator	PE with ceramic coating	1.05	1978[11]
Electrolyte	LiFSI/DMC (1:1.9 by molar)	5.98	133.9[11]
Anode collector	Copper	2.12	385[11]
Cathode collector	Aluminum	1.35	903[11]
Package	Aluminum plastic film	4.38	1000

Supplementary Note 5: Equivalent maximum temperature and equivalent maximum temperature rise rate of full battery

As displayed in Supplementary Table 3, T_3' represents the equivalent maximum temperature of full battery caused by the chemical reactions in the partial cells. $(\mathrm{d}T/\mathrm{d}t)'_{max}$ represents the equivalent maximum temperature rise rate of full battery caused by the chemical reactions in the partial cells, and it is calculated based on Supplementary Equation 3".

$$(\mathrm{d}T/\mathrm{d}t)'_{\mathrm{max}} = (\mathrm{d}T/\mathrm{d}t)_{\mathrm{max}} \cdot \frac{M_{\mathrm{Partial\ cell}}}{M_{\mathrm{Full\ cell}}} \quad \text{(Supplementary Equation 3)}$$

The heat released in AnEly partial cells can heat the full battery to T_3' of 442.7℃ with $(\mathrm{d}T/\mathrm{d}t)'_{max}$ of 501.2℃ s^{-1}, which is in fact a thermal runaway state. That is, the reactions between the anode and the LiFSI-based concentrated electrolyte can bring the full battery to thermal runaway. Similarly, the full cell can be brought to 648.6℃ with a $(\mathrm{d}T/\mathrm{d}t)'_{max}$ of 116.9℃ if the electrolyte stays totally inert. Thus, the reactions between the cathode and the anode can also bring the full battery to thermal runaway at a higher temperature (226.1℃) in comparison with the AnEly cell.

Supplementary Table 3. Equivalent maximum temperature and equivalent maximum temperature rise rate of full battery.

Cells	Mass(g)	T_3'(℃)	$(\mathrm{d}T/\mathrm{d}t)'_{max}$(℃ S^{-1})
CaEly	13.9	257.7	-
AnEly	13.9	442.7	501.2
CaAn	17.5	648.6	116.9

Supplementary Note 6: Normalized Δ*H* comparison of the potential exothermic reactions in the batteries

To better understand the heat contribution of each component to the battery, the discussed Δ*H* in the manuscript was normalized by the total weight of the Ca+An+LiFSI/DMC sample. If the exothermic properties of a single component can be observed, *ΔH* can be normalized by the individual mass. The normalized Δ*H* by the individual component mass and the total mass is listed in Supplementary Tables 4 and 5, for Gr|NMC811 and Gr|NMC532 batteries, respectively.

The exothermic peak of An+LiFSI was placed around 210℃ in this study. In Ref. 40, the exothermic peak was reported at around 200℃. The difference between 210℃ and 200℃ can be attributed to the possible differences in the anode material, the SEI and parameters.

The fully-delithiated NMC811 (Ca) displayed four exothermic peaks between 200℃ and 500℃, where the first was centered at 235.1℃ with a Δ*H* of 79.0 J g^{-1}, and the other three small peaks appeared at 331.2℃, 401.3℃, and 481.3℃, respectively, with a total small Δ*H* of 21.6 J g^{-1} (see Supplementary Table 4). These peaks, which are in the Ca samples, should be attributed to the phase transition of Ca. Ca+LiFSI/DMC sample produced a small exothermic peak (Δ*H* of 40.0 J g^{-1}) around 240℃, while another three exothermic peaks took place at around 300℃ (Δ*H* of 15.4 J g^{-1}), 350℃ (Δ*H* of 74.7 J g^{-1}), and 380℃ (Δ*H* of 128.2 J g^{-1}), which can be related to the phase transition of the cathode and the LiFSI decomposition, indicating that the charged cathode and the concentrated electrolyte clearly did not react before 320℃. The small exothermic peaks that took place at around 240℃ and 300℃ can be related to the phase transition of Ca, while the peak at 350 ℃ can be attributed to the decomposition of LiFSI[26, 27], according to the DSC curves of the LiFSI and LIFSI/DMC samples. The peak around 380℃ may be a result of the reactions between the cathode and the products of the LiFSI decomposition. Given that this study aims to probe the trigger and main reactions during thermal runaway, the uncertainties of these exothermic peaks after 320℃ are almost negligible, as the heat measurements at these peaks are negligible in comparison with the heat at and after T_2. It was indicated that the heat from the Ca+LiFSI/DMC was mostly from the phase transition of the cathode and the LiFSI decomposition, and the charged cathode barely reacted with the concentrated LiFSI/DMC without extra heat release before 320℃. This means that the interaction between cathode and LiFSI/DMC only contributes the small heat, which is consistent with the thermal runaway behavior of the CaEly partial cell. For the full cell, the contribution of Ca+LiFSI/DMC to thermal runaway is hard to say, as it is reasonable that major of Ca and LiFSI/DMC would participate in An+LiFSI/DMC and Ca+An reactions.

Supplementary Table 4. Normalized Δ*H* released by the individual component weight and the total weight of Ca+An+LiFSI/DMC in the Gr|NMC811 sample.

Gr|NMC811 sample	Peak temperature(℃)	Δ*H* normalized by the individual component weigh (J g^{-1})	Δ*H* normalized by total weight of Ca+An+LiFSI/DMC (J g^{-1})
An	288.7	239.5	67.9
An + DMC	289.8	150.4	72.7
LiFSI	352.2	1254.5	250.9
LiFSI + DMC	351.9	729	279.5
An+ LiFSI/DMC	209.6	904.3	602.9
An + LiFSI	210.9	1568	757.9
LiPF6	233.8	-482.4	-20.1
An + LiPF6	213.2	563.5	183.1
Ca	235.1, 331.2, 401.3, 481.3	144 (110.3, 0.36, 26.6, 6.7)	100.6 (79.0, 0.3, 19.1, 2.2)
Ca + LiFSI/DMC	231.9, 297.7, 344.1, 376.0	271.5 (59.2, 33.4, 1.4, 177.5)	194.6 (42.4, 23.9, 1, 127.2)
Ca + An	239.5/279.4	1352.5	834
An + LiFSI/TMP	214.5	810.6	540.4

Supplementary Table 5. Normalized *ΔH* released by the individual component weight and the total weight of Ca+An+LiFSI/DMC in the Gr|NMC532 sample.

Gr|NMC532 sample	Peak temperature (℃)	Δ*H* normalized by the individual component weight (J g^{-1})	Δ*H* normalized by total weight of Ca+An+LiFSI/DMC (J g^{-1})
An	286.8	240.6	68.2
LiFSI	352.2	1254.5	250.9
An+ LiFSI/DMC	209.3	900.5	600.3
An + LiFSI	210.5	1588.5	767.8
Ca	263.3, 419.8	140.3 (85.0, 55.3)	46.8 (28.3, 18.4)
Ca + An	272.1/394.3	1150.2 (879.0, 271.2)	709.3 (542.1, 167.2)

Supplementary Note 7: The interaction between lithiated anode and $LiPF_6$

The thermal stability of $LiPF_6$ and its mixture with the lithiated anode (An+$LiPF_6$) were also investigated by DSC and TGA, as shown in Supplementary Fig. 3. In comparison, the data of LiFSI and An+LiFSI in Fig. 4a were shown as well. It was observed that $LiPF_6$ displayed two endothermic peaks at 195.6℃ and 233.8℃, respectively (see Supplementary Fig. 3a). The first small peak was related to the melting of $LiPF_6$, and the second peak was attributed to the decomposition of $LiPF_6$ ($LiPF_6$ (s) ↔ LiF (s) + PF_5 (g))[8, 9]. The second endothermic peak coincides with the TGA curve of $LiPF_6$, which showed a substantial decrease of 70% around 233.8℃ due to the decomposition of $LiPF_6$[29, 30] (see Supplementary Fig. 3b). The endothermic behavior of $LiPF_6$ was completely different from that of LiFSI, and LiFSI showed an intensive exothermic peak around 350℃. The An+$LiPF_6$ sample showed a mild exothermic peak and weight loss at 213.2℃ with a ΔH of 183.1 J g^{-1}. However, the heat from An+$LiPF_6$ was far lower than that released by the An+LiFSI sample (757.9 J g^{-1}). From these results, it can be concluded that there was an intensive exothermic reaction between the FSI^--based salt and the lithiated anode. The tremendous heat from the An+LiFSI sample was proven to directly throw the Gr|NMC811 battery into a thermal runaway. As for the An+$LiPF_6$ sample in the Gr|NMC811 battery, the released and relatively small heat was probably not the main exothermic reaction in the system. At present, the thermal runaway mechanism of the conventional electrolyte in the Gr|NMC811 battery is still not clear. However, based on the above analysis, it can be concluded that $LiPF_6$ might not be involved in the trigger or main reactions of the thermal runaway.

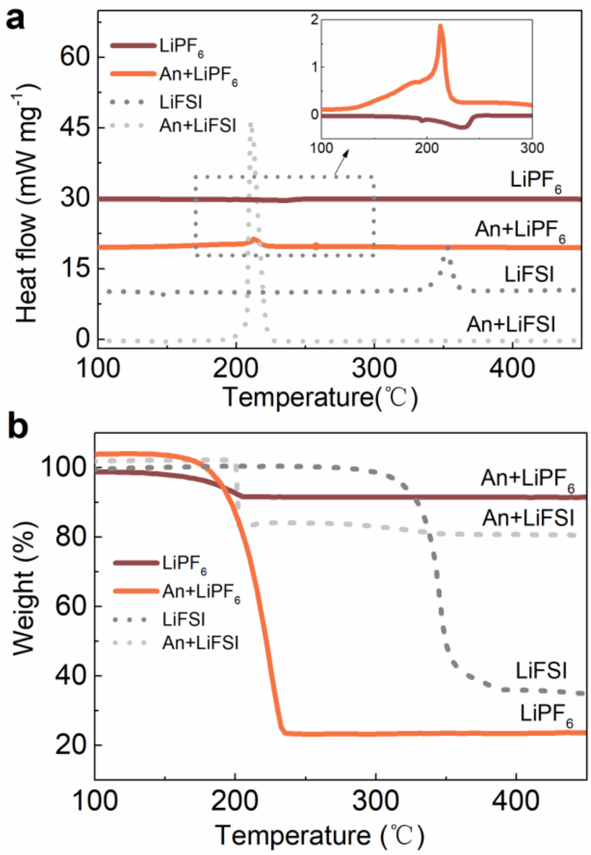

Supplementary Fig. 3 a DSC traces. **b** TGA curves of $LiPF_6$ and its mixture with the lithiated anode (An+ $LiPF_6$) (In contrast, LiFSI and An+LiFSI in Fig.4a were uploaded as well).

Supplementary Note 8: DSC traces of Ca+Separator

The samples of Separator and Ca+Separator were prepared and the DSC traces were displayed in Supplementary Fig. 4. For Ca+Separator, two endothermic peaks at 140.5℃ and 480.2℃, respectively, which were related to the characters of the separator. The exothermic peak at around 240℃ with Δ*H* of 135.4 J/g, which was slightly higher than Δ*H* of Ca individually with a value of 100.6 J g^{-1} at 235.1℃. This would attribute to the interaction between cathode and separator. The Δ*H* of Ca + Separator was much smaller than An+LiFSI/DMC and Ca+An, which Ca + Separator can be deduced to have a small heat contribution to thermal runaway.

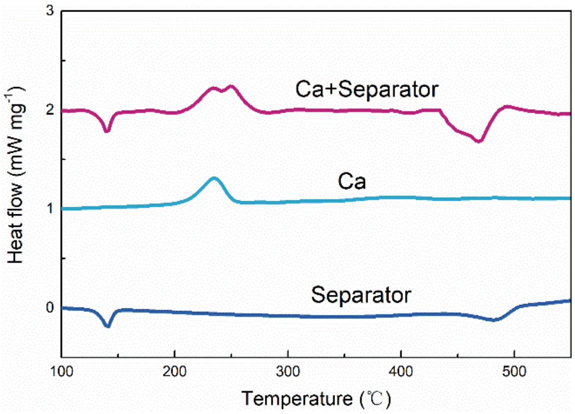

Supplementary Fig. 4 DSC traces of cathode, separator and Ca+Separator

Supplementary Note 9: XPS analysis of An+LiFSI sample

Supplementary Fig. 5 displayed the XPS results of An+LiFSI sample. For the F 1s spectra, the peak at 684.50 eV indicated the presence of LiF. It has been reported that LiF was resulted from the reaction of FSI^- anion with the reductive LiC_6[10]. For the S 2p spectra, the peak at 164.4 eV was suggested to be elemental sulfur[11], involving the redox mechanism between the oxidative FSI^- and the reductive LiC_6 at a high temperature. Another two peaks can be related to the SO-related bonds, such as the Li_2SO_3 (166.7 eV) and Li_2SO_4 (170.2 eV), as the reduced byproducts of FSI^-[12-15]. Actually, the Li_2SO_3 and Li_2SO_4 were reported to be present in the SEI layers as the electrochemically reduced products of the LiFSI salt [10, 16, 17]. The S 2p spectra revealed evidence of the detachment and reaction of the SO_2 group from the breakdown of the S-N bond in the FSI^-[18]. As for the C 1s spectra , the peak at 284.8 eV can be attributed to the C-C bonds, and the peak at 290.0 eV corresponds to the C = O bonds, which confirms the existence of Li_2CO_3[12, 19].

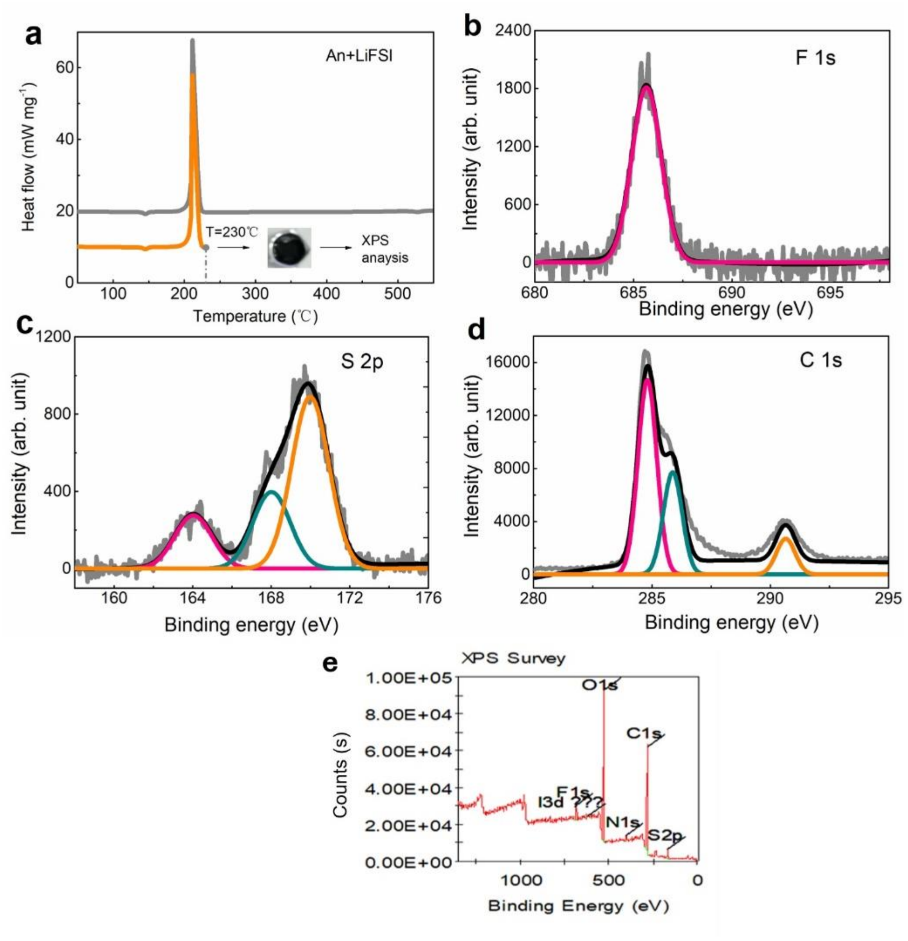

Supplementary Fig. 5 a DSC measurement of the sample with lithiated anode and LiFSI was ceased at 230℃. **b** – **d** XPS analysis of the byproducts of An + LiFSI. **e** The full XPS spectrum of An + LiFSI

Supplementary Note 10: XPS analysis of An+LiFSI/TMP sample

Similar to An+LiFSI sample, the byproducts of An+LiFSI/TMP sample were also prepared and characterized, as shown in Supplementary Fig. 6. For the F 1s spectra, the same peak at 684.50 eV with An+LiFSI sample indicated the presence of LiF due to LiFSI reduction by the anode, and the peak at 687.9 eV was related to the residue of LiFSI[20]. For the S 2p spectra, the peaks were suggested to be the SO-related bonds, such as the Li_2SO_3 (167.3 eV) and Li_2SO_4 (168.6 eV, 169.7 eV)[21], as the reduced byproducts of FSI^-. As for the C 1s spectra, the peak at 284.8 eV attributed to the C-C bonds. The peak at 288.8 eV corresponded to the C = O bonds, which confirmed the existence of Li_2CO_3[22], and the peak at 283.0 eV was attributed to Li_xC_6[20]. These XPS results of An+LiFSI/TMP revealed that the chemical interaction between lithiated anode and LiFSI around 210℃. It should be noted that, for the P 2p spectra, there were no peaks to be detected, it can be deduced that TMP would not involve in the chemical reaction between anode and electrolyte due to the full volatilization of TMP around 210℃ or the phosphorus was a trace element in the sample

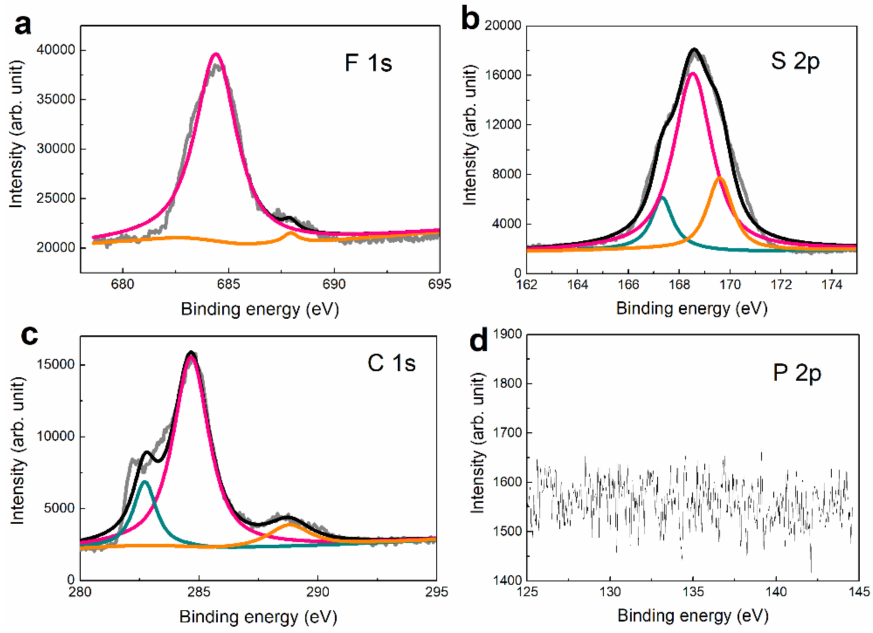

Supplementary Fig. 6 a – d XPS analysis of the byproducts of An + LiFSI/TMP.

Supplementary Note 11: Details of Gr|NMC811 and Gr|NMC532 batteries

As shown in Supplementary Table 6, the sizes of the Gr|NMC811 and Gr|NMC532 batteries are both 58mm×68mm×2.5mm. The areal capacity (*C*) of the cathode and the anode in Gr|NMC811 is 2.80 and 3.22 mAh cm^{-2}, whereas it is 2.75 and 3.22 mAh cm^{-2} in Gr|NMC532, respectively. Three kinds of electrolytes (concentrated LiFSI/DMC, concentrated LiFSI/TMP, and 1M $LiPF_6$ in EC:EMC) were injected into Gr|NMC811, and their safety properties were examined. To fully investigate the electrolyte performance in the batteries, Gr|NMC532 batteries were also employed, and the electrolytes of the concentrated LiFSI/DMC and 1M $LiPF_6$ in EC:EMC were injected into their cells for the formation. After that, the cells were cycled in the voltage range of 2.85–4.2 V at 1/3C under 25℃ for two cycles. Then, the cells were fully charged to 4.2V for the safety measurements.

Supplementary Table 6. Energy density of the battery and the areal capacity (*C*) of the electrodes.

Parameter	Gr|NMC811 battery	Gr|NMC532 battery
Capacity (Ah)	0.93	1.2
Energy density (Wh kg^{-1})	191	182
Cathode c_s (mAh cm^{-2})	2.80	2.75
Anode c_s (mAh cm^{-2})	3.22	3.22
Electrolyte	LiFSI/DMC (1:1.9 by molar)<br>LiFSI/TMP (1:1.9 by molar)<br>1M $LiPF_6$ in EC:EMC (3:7 by volume)	LiFSI/DMC (1:1.9 by molar)<br>1M $LiPF_6$ in EC:EMC (3:7 by volume)
Separator	PE with ceramic coating	PE with ceramic coating
Battery size (mm × mm × mm)	58 × 68 × 2.5	58 × 68 × 2.5

Supplementary Note 12: ARC tests on the partial cells and lateral heating tests

The fabrication process of the AnEel, CaEle, and CaAn partial cells is shown in Supplementary Fig. 7a, where this process was conducted in an argon-filled glove box in which the oxygen and water contents were controlled below 0.1 ppm. After fabrication, the partial cells were weighted, the mass is 13.9g, 13.9g, 17.5g for AnEel, CaEle, and CaAn partial cells, respectively. Then, the partial cells were transferred to the drying room for thermocouple insertion and then resealed with a silicone sealant, which can maintain tightness and elasticity even at 343℃. After that, the cells were let to stand still for 24h for the silicone sealant to solidify. Finally, ARC tests were performed on the cells. The lateral heating test process is shown in Supplementary Fig. 7b. The ceramic heat was fixed at the surface of the full charge state of the battery by a Teflon tape with a heat resistance of 260℃. Then, the battery and the ceramic heat were clamped together with two epoxy plates. After that, a DC power with 20W started heating the battery until the inner temperature reached 200℃, where the temperature was recorded by the built-in micro-thermocouple. Then, the behavior of the battery was recorded by the video. After the test, the epoxy plates and a part of the Teflon tape were kept intact. Flame test and lateral heating test indicate that the battery decomposition products may or may not support a diffusion flame in quiescent air, which cannot provide a quantitative assessment of energy release in flaming combustion. Additionally, the flaming combustion of ejected battery materials is known to significantly speed up thermal runaway propagation in battery packs[23], and TMP would reduce the propagation speed. The influence of the concentrated LiFSI/TMP electrolyte on propagation performance needs more investigations.

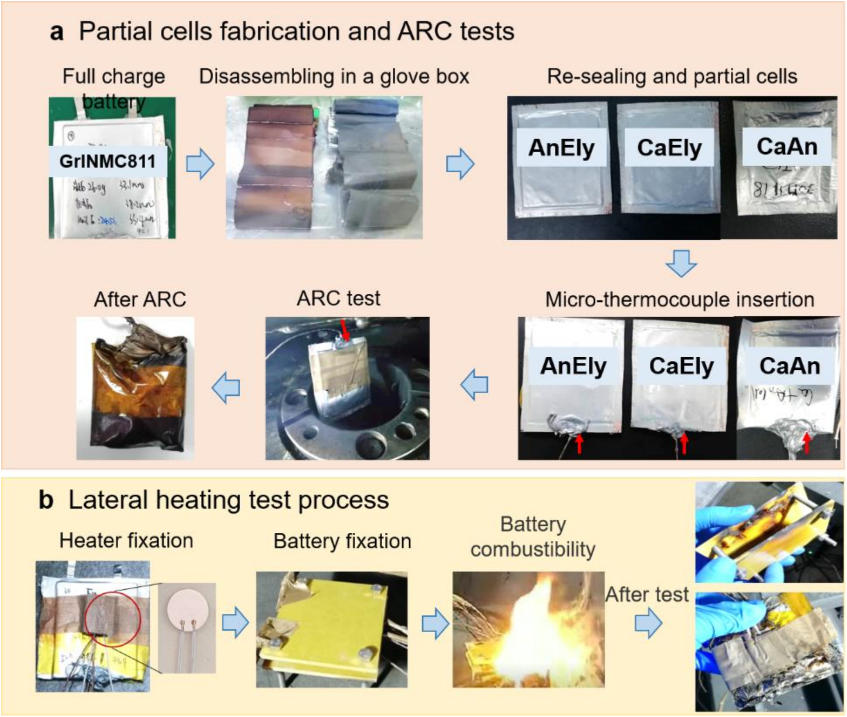

Supplementary Fig. 7 **a** AnEel, CaEle, and CaAn partial cells fabrication and ARC tests. **b** The lateral heating tests on the batteries to examine the combustibility features.

Supplementary References

1. Hess, S., Wohlfahrt-Mehrens, M. & Wachtler, M. Flammability of Li-Ion Battery Electrolytes: Flash Point and Self-Extinguishing Time Measurements. *Journal of the Electrochemical Society* 162, A3084-A3097 (2015).
2. Wang, J. et al. Fire-extinguishing organic electrolytes for safe batteries. *Nature Energy* 3, 22-29 (2017).
3. Feng, X.N. et al. Thermal runaway features of large format prismatic lithium ion battery using extended volume accelerating rate calorimetry. *Journal of Power Sources* 255, 294-301 (2014).
4. Liu, X. et al. Thermal Runaway of Lithium-Ion Batteries without Internal Short Circuit. *Joule* 2, 2047-2064 (2018).
5. Coman, P.T., Darcy, E.C., Veje, C.T. & White, R.E. Modelling Li-Ion Cell Thermal Runaway Triggered by an Internal Short Circuit Device Using an Efficiency Factor and Arrhenius Formulations. *Journal of the Electrochemical Society* 164, A587-A593 (2017).
6. Coman, P.T., Darcy, E.C., Veje, C.T. & White, R.E. Numerical analysis of heat propagation in a battery pack using a novel technology for triggering thermal runaway. *Applied Energy* 203, 189-200 (2017).
7. Feng, X.N. et al. A Coupled Electrochemical-Thermal Failure Model for Predicting the Thermal Runaway Behavior of Lithium-Ion Batteries. *Journal of the Electrochemical Society* 165, A3748-A3765 (2018).
8. Lekgoathi, M.D.S., Vilakazi, B.M., Wagener, J.B., Le Roux, J.P. & Moolman, D. Decomposition kinetics of anhydrous and moisture exposed LiPF6 salts by thermogravimetry. *Journal of Fluorine Chemistry* 149, 53-56 (2013).
9. Choi, N.-S., Profatilova, I.A., Kim, S.-S. & Song, E.-H. Thermal reactions of lithiated graphite anode in LiPF6-based electrolyte. *Thermochimica Acta* 480, 10-14 (2008).
10. Wang, M. et al. Effect of LiFSI Concentrations To Form Thickness- and Modulus-Controlled SEI Layers on Lithium Metal Anodes. *The Journal of Physical Chemistry C* 122, 9825-9834 (2018).
11. Fu, Y. & Manthiram, A. Orthorhombic Bipyramidal Sulfur Coated with Polypyrrole Nanolayers As a Cathode Material for Lithium–Sulfur Batteries. *The Journal of Physical Chemistry C* 116, 8910-8915 (2012).
12. Chen, X. et al. Facile synthesis of Li2S@C composites as cathode for Li–S batteries. *Journal of Energy Chemistry* 37, 111-116 (2019).
13. Liu, J., Nara, H., Yokoshima, T., Momma, T. & Osaka, T. Carbon-coated Li2S Synthesized by Poly(vinylpyrrolidone) and Acetylene Black for Lithium Ion Battery Cathodes. *Chemistry Letters* 43, 901-903 (2014).
14. Zhan, Y., Yu, H., Ben, L., Chen, Y. & Huang, X. Using Li2S to Compensate for the Loss of Active Lithium in Li-ion Batteries. *Electrochim Acta* 255, 212-219 (2017).
15. Zhang, S.S. A cost-effective approach for practically viable Li-ion capacitors by using Li2S as an in situ Li-ion source material. *Journal of Materials Chemistry A* 5, 14286-14293 (2017).
16. Feng, X. et al. Understanding the degradation mechanism of rechargeable lithium/sulfur cells: a comprehensive study of the sulfur-graphene oxide cathode after discharge-charge cycling. *Phys Chem Chem Phys* 16, 16931-16940 (2014).
17. Xu, C. et al. Interface layer formation in solid polymer electrolyte lithium batteries: an XPS study. *J. Mater. Chem. A* 2, 7256-7264 (2014).
18. Parikh, P. et al. Role of Polyacrylic Acid (PAA) Binder on the Solid Electrolyte Interphase in Silicon Anodes. *Chemistry of Materials* 31, 2535-2544 (2019).
19. Michan, A.L. et al. Fluoroethylene Carbonate and Vinylene Carbonate Reduction: Understanding Lithium-Ion Battery Electrolyte Additives and Solid Electrolyte Interphase Formation. *Chemistry of Materials* 28,

8149-8159 (2016).

20. Eshetu, G.G. et al. In-Depth Interfacial Chemistry and Reactivity Focused Investigation of Lithium-Imide- and Lithium-Imidazole-Based Electrolytes. *Acs Appl Mater Inter* 8, 16087-16100 (2016).
21. Zhan, Y.J., Yu, H.L., Ben, L.B., Chen, Y.Y. & Huang, X.J. Using Li2S to Compensate for the Loss of Active Lithium in Li-ion Batteries. *Electrochim Acta* 255, 212-219 (2017).
22. Liu, X.B. & Liu, J.P. Effect of air humidity on microstructure and phase composition of lithium deuteride corrosion products. *Corros Sci* 115, 129-134 (2017).
23. Lee, C., Said, A.O. & Stoliarov, S.I. Passive Mitigation of Thermal Runaway Propagation in Dense 18650 Lithium Ion Cell Assemblies. *Journal of the Electrochemical Society* 167 (2020).
