# Supplementary material for: Thermal runaway of Lithium-ion batteries employing LiN(SO2F)2-based concentrated electrolytes
Source: Nat Commun. 2020 Oct 9;11:5100. doi: 10.1038/s41467-020-18868-w (PMC7547674; doi:10.1038/s41467-020-18868-w)
Supplement: Supplementary file 2 — Description of Additional Supplementary Files [file 41467_2020_18868_MOESM2_ESM.pdf]

Description of Additional Supplementary Files

File Name: Supplementary Movie 1
Description: The combustibility of Gr|NMC811 battery with concentrated LiFSI/DMC electrolyte in the lateral heating test.

File Name: Supplementary Movie 2
Description: The combustibility of Gr|NMC811 battery with concentrated LiFSI/TMP electrolyte in the lateral heating test.
